# Effect of Virtual Reality Relaxation on Alarm Fatigue and Professional Quality of Life Among Nurses in Intensive Care Units

**DOI:** 10.1002/hsr2.73355

**Published:** 2026-10-10

**Authors:** Fatemeh Mirzahosseini, Mahlagha Dehghan, Moazame Jazinizade, Neda Asadi

**Affiliations:** ^1^ Student Research Committee Kerman University of Medical Sciences Kerman Iran; ^2^ HIV/STI Surveillance Research Center and WHO Collaborating Center for HIV Surveillance, Institute for Futures Studies in Health Kerman University of Medical Sciences Kerman Iran; ^3^ Reproductive and Family Health Research Center Kerman University of Medical Sciences Kerman Iran; ^4^ Nursing Research Center Kerman University of Medical Sciences Kerman Iran

**Keywords:** alarm fatigue, nursing, professional quality of life, virtual reality

## Abstract

**Introduction:**

Intensive care unit nurses are faced with a large number of equipment alarms daily, which can increase the likelihood of errors and threaten their professional quality of life. Today, the use of virtual reality to create relaxing experiences in stressful environments has received attention. This study aimed to determine the effect of virtual reality relaxation on alarm fatigue and the professional quality of life among ICU nurses.

**Methods:**

This is an intervention study with a pretest‐posttest design with a control group. ICU nurses who met the inclusion criteria were selected conveniently and were randomly assigned to two intervention and control groups (30 people in each group). The intervention was implemented as three 20‐min virtual reality relaxation sessions over a week. The data collection tools included the Alarm Fatigue Scale (AFS) and the Professional Quality of Life (ProQOL) questionnaire. The questionnaires were completed before and immediately after the intervention.

**Results:**

The results showed that the mean AFS score improved significantly after the intervention (*p* < 0.001), and there was a significant difference between the intervention and control groups (*p* = 0.014). The mean dimensions of ProQOL also improved significantly in all three dimensions of compassion satisfaction, burnout, and secondary trauma stress after the intervention (*p* < 0.001). There was a significant difference between two groups in all dimensions after the intervention (*p* < 0.001).

**Conclusion:**

The significant improvement in AFS and ProQOL scores indicates that the use of new technologies, such as virtual reality can be considered as a complementary tool in promoting the mental and professional health of nurses in stressful environments.

## 1 Introduction

The complexity of caring for patients admitted to ICUs is significantly higher than in other departments. The presence of multiple equipment is an integral part of the intensive care unit. To keep patients safe, clinical alarms are used to detect changes in the patient's condition [1, 2].

However, devices may generate a large number of alarms, which can lead to alarm fatigue in nurses [3]. Alarm fatigue, a condition in which caregivers become indifferent to alarms due to prolonged exposure to them, is one of the most important health technology hazards [4].

This can cause a range of physical, psychological, and interpersonal problems [5]. Therefore, it is essential to pay close attention to the nature of ICU nurses' work and the effects of alarm fatigue on their professional quality of life [6]. Environments with high levels of stress have a significant impact on the professional quality of life among nurses [2]. In recent years, the use of virtual reality relaxation (VRelax) in care settings has received attention because it leads to increased positive emotions [7]. Vázquez et al. suggested that virtual reality could be considered an alternative to meditation in the ICU. The virtual reality experience, regardless of time and place, can allow people to forget that they are in the ICU [8].

According to the researchers' reviews, perceived stress of ICU nurses [8], sleep quality of ICU patients [9], and stress of caregivers [10] were the relevant studies found. No study was found that examined the effect of VRelax on nurses' professional quality of life and the consequences of alarm fatigue. Considering the complex working conditions of nurses in the ICU and the stress caused by noise from ward equipment, and on the other hand, paying attention to new and practical interventions, it seems that addressing this study is of particular importance, because such cases subsequently help improve the quality of patient care. This study will be conducted to determine the effect of VRelax on alarm fatigue and the professional quality of life among ICU nurses.

## 2 Material and Methods

This interventional study employed a pretest–posttest design with a control group and was conducted among intensive care unit nurses in southeastern Iran in 2025. Eligible nurses were recruited using convenience sampling based on predefined inclusion criteria. To ensure rigorous randomization and allocation concealment, prepared 60 sealed, opaque envelopes containing the group assignment (1:1 allocation ratio). Eligible participants were randomly assigned to the intervention or control groups by selecting an envelope sequentially, only after they had provided informed consent and completed the baseline assessment. A total of 60 nurses participated in the study, with 30 nurses assigned to each group. Inclusion criteria: Having at least a bachelor's degree in nursing, having at least 6 months of work experience in intensive care units, not suffering from neurological and psychiatric diseases, epilepsy, and migraines based on self‐declaration, not having hearing impairment, and not being sensitive to light based on self‐declaration, and not needing to use any types of prescription glasses. Exclusion criteria: Absence from at least one of the intervention sessions, failure to respond to more than 10% of the questions, unwillingness to continue cooperation.

### 2.1 Sample Size Calculation

The required sample size was determined a priori using G*Power software for comparison of two independent means. A two‐sided significance level of 0.05, statistical power of 80%, an equal allocation ratio (1:1), and a medium‐to‐large standardized effect size (Cohen's d = 0.81) were assumed. Based on these parameters, a minimum of 25 participants was required in each group. To account for an anticipated attrition rate of approximately 17%, the sample size was increased to 30 participants per group, resulting in a total target sample of 60 participants. Because the intervention involved virtual reality, participants and the intervention provider could not be blinded. The statistician was blinded to group allocation during data analysis.

### 2.2 Instrument

Data were collected using three questionnaires, including sociodemographic questionnaire, the alarm fatigue scale (AFS) and the professional quality of life questionnaire (ProQOL).

Sociodemographic questionnaire including: age, gender, marital status, education level, work experience in the ICU, previous experience using virtual reality, and completion of a training course on managing alarms.

### 2.3 The AFS

#### 2.3.1 AFS

Alarm fatigue was assessed using the AFS, developed and validated by Dehghan et al. [11] for assessing alarm fatigue among nurses. The scale consists of 10 items grouped into two subscales: psychosomatic symptoms and care‐related symptoms. Items are rated on a five‐point Likert scale ranging from 0 (never) to 4 (always), yielding a total score ranging from 0 to 40, with higher scores indicating greater alarm fatigue. Based on the proposed scoring criteria, scores of 0–10, 11–20, 21–30, and 31–40 indicate no/little, mild, moderate, and moderately severe alarm fatigue, respectively. The original validation study demonstrated good internal consistency, with Cronbach's α values ranging from 0.85 to 0.875 for the total scale and its subscales, and item–total correlations ranging from 0.50 to 0.66 [11]. The Persian version of the AFS, which was developed and validated among Iranian nurses by Dehghan et al. [11], was used in this study.

### 2.4 The Professional Quality of Life Questionnaire (ProQOL)

The ProQOL developed by Stamm18 consisted of 30 items and three subscales: compassion satisfaction, burnout, and secondary trauma stress. Items 1, 4, 15, 17, and 29 were scored inversely with a 5‐point Likert scale ranging from 1 (never) to 5 (often). Each subscale is independent, and subscale scores were not added. Scores ≤ 22 indicate low CS, lower burnout, or lower secondary trauma stress, scores 23−41 indicate moderate, and scores > 42 indicate higher CS, more burnout, or more traumatic stress. To evaluate the instrument reliability by the internal consistency method, Stamm calculated Cronbach's ⍺ coefficients of the CS (0.88), burnout (0.75), and secondary trauma stress (0.81) [12]. The Persian translation and psychometric properties of the ProQOL have been evaluated among healthcare providers in Iran by Ghorji et al. [13]. In the present study, the 30‐item Persian version of the ProQOL was used. The Cronbach's ⍺ of 0.73 using internal consistency [13].

### 2.5 Intervention

The intervention was delivered using Shinecon SC‐G06EB virtual reality (VR) glasses. The VR intervention consisted of three sessions delivered over 1 week, with each session lasting 20 min. Before the first session, participants received standardized verbal and practical instructions from the researcher regarding the use of the VR glasses, including how to wear and adjust the device, how to maintain a comfortable viewing position, and how to report any discomfort during the session. The same instructions were provided to all participants to ensure consistency in intervention delivery.

The VR content was delivered through the VRelax application and consisted of immersive 360‐degree natural environments designed to promote relaxation. The environments included relaxing scenes such as waterfalls, lakes, forests, and sea waves. The videos were presented at a resolution of 1080 × 720 pixels. The same predefined VR content and exposure duration were used for all participants in the intervention group, and no participant was permitted to select alternative content. This procedure was used to minimize variability in the intervention and ensure that participants were exposed to comparable VR stimuli. The VR experience incorporated both visual and auditory stimuli. Participants viewed the 360‐degree natural environments while the accompanying audio was delivered through the VR device. The audio component was kept consistent across participants by using the same predefined content and playback settings for each session. Participants were instructed to remain seated and avoid unnecessary movement during the intervention. Sessions were conducted in a quiet and safe setting to minimize external sensory distractions and interruptions.

To ensure temporal standardization, the intervention was administered during the nurses' scheduled break and only after at least one‐third of their work shift had elapsed. Each participant received the intervention under comparable conditions with respect to session duration, VR equipment, content, and timing within the work shift. The researcher remained available throughout the sessions to monitor adherence to the intervention protocol and address any technical or participant‐related problems. Potential VR‐related adverse effects were considered before and during each session. Participants were informed that they should immediately report symptoms such as dizziness, nausea, headache, eye strain, visual discomfort, disorientation, or other discomfort associated with VR exposure. If such symptoms occurred, the intervention would be interrupted, and the participant would be allowed to remove the VR glasses and rest until the symptoms resolved. The occurrence of any adverse symptoms was monitored by the researcher throughout the intervention period.

To minimize the risk of cross‐contamination between participants, the VR glasses were disinfected with a diluted alcohol solution after each use. The equipment was inspected before each session to ensure proper functioning and appropriate positioning of the device. Participants assigned to the control group did not receive the VR intervention and followed their usual rest routine during the corresponding 20‐min period. No additional relaxation intervention was provided to the control group during this period. Alarm fatigue and professional quality of life were assessed in both groups using the study questionnaires at baseline, before initiation of the intervention, and again after completion of the three‐session intervention.

### 2.6 Data Analysis

Data were analyzed using SPSS version 25. Descriptive statistics were used to summarize participant characteristics and study variables. Baseline characteristics were compared between groups using independent‐samples *t*‐tests, chi‐square tests, or Fisher's exact tests, as appropriate. ANCOVA was used to compare post‐intervention outcomes between groups while controlling for baseline scores. Statistical assumptions for ANCOVA were assessed before analysis. Statistical significance was set at *p* < 0.05.

### 2.7 Ethical Considerations

Ethical approval for this study was obtained from the Ethics Committee of Kerman University of Medical Sciences (R. KMU. REC.1403.265) before data collection began. Written informed consent was obtained from all participants. ICU nurses were assured that their information would be kept strictly confidential. Furthermore, participants were informed of their right to withdraw from the study at any time without this decision affecting the patient's care or treatment.

## 3 Results

Table 1 presents the comparison of baseline demographic and clinical characteristics between the intervention and control groups. Initial analysis revealed no statistically significant difference in age, gender, marital status, educational level, previous experience using virtual reality, complete a training course on manage alarms, age and work experience between the two groups.

**Table 1:** The demographic and background characteristics of ICU nurses.

Group variable	Intervention (*n* = 30)		Control (*n* = 30)		Statistical test	*p*‐value
	Frequency	Percent	Frequency	Percent		
Sex						
Female	19	63.3	20	66.7	0.073 = 2 χ	0.99
Male	11	36.7	10	33.3		
Marital status						
Single	8	26.7	11	36.7	4.501 = Fisher's Exact test	0.120
Married	18	60.0	19	63.3		
Widow	4	13.3	0	0		
Educational level						
Bachelor	24	80.0	25	83.3	0.111 = 2 χ	0.739
Master and above	6	20.0	5	16.7		
Previous experience using virtual reality						
Yes	1	3.3	2	6.7	0.351 = 2 χ	0.99
No	29	96.7	28	93.3		
Complete a training course on manage alarms						
Yes	2	6.7	2	6.7	—	—
No	28	93.3	28	93.3		

	Mean	SD	Mean	SD	Independent *t*‐test	*p*‐value
Age	31.97	5.80	31.30	5.42	0.460	0.667
Work experience (y)	5.32	3.32	4.60	3.39	0.827	0.412

According to Table 2, the mean total alarm fatigue score in the intervention group decreased from 18.23 before the intervention to 12.90 after the intervention, which was statistically significant. In the control group, the mean total alarm fatigue score decreased from 17.43 before the intervention to 17.14 after the intervention, but this was not statistically significant. There was a significant difference between the intervention and control groups in terms of alarm fatigue score after the intervention. There was a significant difference between the two groups after the intervention in the score of psychosomatic symptoms. Also, by performing the ANCOVA test and controlling for the pre‐intervention score, care‐related symptoms, there was still a significant difference between the intervention and control groups. (*p* < 0.001, F = 11.428, Effect size = 0.196).

**Table 2:** The mean (SD) Alarm fatigue score of ICU nurses before and after the intervention.

Alarm fatigue dimension	Time	Intervention Mean ± SD	Control Mean ± SD	Independent *t*‐test, *p*‐value	Within‐group *p*‐value*	ANCOVA F (*p*‐value)	Partial η^2^	Adjustedmean difference (95% CI)
Psychosomatic symptoms	Before	7.80 ± 3.98	8.30 ± 3.87	−0.493, 0.624	—	—	—	—
	After	4.63 ± 3.13	7.93 ± 3.94	−3.561, 0.001	< 0.001 0.125	10.82 (< 0.001)	0.161	−3.24 (−4.48 to −2.00)
Care‐related symptoms	Before	10.43 ± 3.28	9.13 ± 3.97	1.383, 0.172	—	—	—	—
	After	8.27 ± 3.17	9.21 ± 4.24	−0.966, 0.338	< 0.001 0.636	11.428 (< 0.001)	0.196	−2.14 (−3.42 to −0.86)
Total alarm fatigue score	Before	18.23 ± 5.34	17.43 ± 6.69	0.512, 0.611	—	—	—	—
	After	12.90 ± 5.31	17.14 ± 7.36	−2.543, 0.014	< 0.001 0.504	8.76 (0.004)	0.134	−4.79 (−7.07 to −2.51)

According to Table 3, although there was no significant difference between the intervention and control groups after the intervention in terms of the score of compassion satisfaction, by conducting the ANCOVA test and controlling for the score before the intervention, there was a significant difference between the intervention and control groups (*p* = 0.009, F = 7.437, Effect size = 0.119). The results showed that there was a significant difference between the intervention and control groups after the intervention in the scores of burnout. A significant baseline difference was observed between the intervention and control groups in secondary traumatic stress scores (Table 3). To account for this baseline imbalance, the pre‐intervention secondary traumatic stress score was included as a covariate in the ANCOVA. After adjustment for the baseline score, a significant difference remained between the intervention and control groups in post‐intervention secondary traumatic stress scores (F = 14.725, *p* < 0.001, Effect size = 0.208).

**Table 3:** The mean (SD) Professional quality of life score of ICU nurses before and after the intervention.

Professional quality of life dimension	Time	Intervention Mean ± SD	Control Mean ± SD	Independent *t*‐test/Mann–Whitney, *p*‐value	Within‐group *p*‐value*	ANCOVAF (*p*‐value)	Partial η^2^	Adjustedmean difference (95% CI)
Compassion satisfaction	Before	20.63 ± 6.09	21.73 ± 5.21	−0.751, 0.455	—	—	—	—
	After	23.13 ± 6.17	21.89 ± 5.40	0.812, 0.420	0.002/0.934	7.437 (0.009)	0.119	1.84 (0.46 to 3.22)
Burnout	Before	18.48 ± 4.63	19.73 ± 2.65	−1.312, 0.189	—	—	—	—
	After	15.10 ± 5.22	19.93 ± 2.79	−3.309, 0.001	< 0.001/0.347	9.624 (0.003)	0.143	−4.36 (−6.25 to −2.47)
Secondary traumatic stress	Before	13.93 ± 5.28	17.97 ± 4.94	−3.055, 0.003	—	—	—	—
	After	11.13 ± 5.78	18.00 ± 4.88	−4.923, < 0.001	0.001/0.990	14.725 (< 0.001)	0.208	−6.42 (−8.83 to −4.01)

## 4 Discussion

The findings suggest that VR relaxation was associated with lower alarm fatigue and favorable changes in some dimensions of professional quality of life among ICU nurses.

The results from the intervention group in this study are effectively consistent with previous reports on the reduction of psychological burden in ICU nurses through VR. In particular, the study results are consistent with the evidence in the scientific literature and confirm the positive effects of VRelax interventions in stressful healthcare environments. Bodet et al. [14] showed that even a short VR session can reduce fatigue in ICU nurses and make rest time more efficient. This is consistent with the overall reduction in AFS in the present study [14]. Also, the findings of the present study on stress reduction are consistent with the results of Nijland et al. [9], who reported a 39.9% reduction in the perceived stress levels of intensive care nurses after VR relaxation sessions [15].

Riches et al.'s systematic review provided evidence of the short‐term effectiveness of virtual reality relaxation for promoting relaxation and reducing stress, providing a solid theoretical basis for observing a similar effect on alarm fatigue [15]. The specific findings of this study provide preliminary evidence of a potential beneficial effect of VR‐based relaxation on alarm fatigue. Specifically, the largest reduction in AFS was related to psychosomatic symptoms, suggesting a direct and profound effect of VR on nurses' physiological stress responses. This finding is stronger than the effect observed in the care‐related symptoms dimension.

In contrast, the only study in the reviewed literature that showed a significant difference in results was Meese et al. [10], who did not observe a significant change in cognitive fatigue indices after using virtual reality simulation in clinical training [10]. The main difference is in the nature of the intervention. While the current study focused on relaxation, the primary goal of which is to reduce environmental stress responses (such as alarm fatigue), Meese's study focused on educational simulation. Educational interventions, while they may increase cognitive load during simulation, do not necessarily lead to reduced fatigue or chronic workplace stress. The second factor is the difference in VR content; since many successful VR relaxation interventions rely on nature‐based stimuli (as Riches et al. [15] noted), the absence of such components in the Meese educational environment could be a reason for the lack of positive effects [15].

In the area of professional quality of life, the VR intervention was able to significantly reduce burnout and secondary traumatic stress, suggesting potentially favorable changes in selected dimensions of professional quality of life on professional mental health. The increase in compassion satisfaction also confirms that the intervention environment not only reduced stress but also improved the quality of professional interactions [16].

The validity of these results is strengthened by the observation of demographic similarities between the two groups with no significant differences in age, gender, work experience, and shift work, indicating that the findings were observed despite the absence of statistically significant baseline differences in the measured demographic characteristics between the two groups and not due to underlying background differences between participants. Furthermore, the results of the improvement in the dimensions of professional quality of life are consistent with the findings of Abbasi et al. on the positive impact of VR on the quality of work life of employees [17], as well as Lee et al. [18] on the improvement of sleep quality in ICU patients, indicating the ability of VR to modulate environmental‐psychological stimuli in the ICU [19].

Several limitations should be considered when interpreting the findings. The relatively small sample size and recruitment of participants from a limited group of intensive care unit nurses may have reduced the representativeness of the sample and limited the generalizability of the findings beyond similar clinical settings. Furthermore, the intervention consisted of only three VRelax sessions delivered over 1 week, and outcomes were assessed immediately following the intervention. As a result, the study design does not allow conclusions to be drawn about the persistence of the observed effects over longer periods. Larger studies with longer intervention periods and adequate follow‐up assessments are needed to determine the durability of effects and further evaluate the potential role of VR as a supportive strategy for managing alarm‐induced fatigue and professional quality of life among nurses.

## 5 Conclusion

A brief VR‐based relaxation intervention using VRelax was associated with reduced alarm fatigue and favorable changes in selected dimensions of professional quality of life among ICU nurses. The findings suggest that VR‐based relaxation may represent a feasible supportive approach that can be incorporated into nurses' break periods. However, its use should be considered as a supportive measure rather than a substitute for organizational strategies.

## Author Contributions

**Fatemeh Mirzahosseini:** conceptualization, investigation, visualization. **Mahlagha Dehghan:** investigation, validation. **Moazame Jazinizade:** investigation, methodology. **Neda Asadi:** conceptualization, writing – original draft, visualization, formal analysis.

## Funding

The authors have nothing to report.

## Ethics Statement

Ethical approval for this study was obtained from the Ethics Committee of Kerman University of Medical Sciences (R. KMU. REC.1403.265) before data collection began. Written informed consent was obtained from all participants. ICU nurses were assured that their information would be kept strictly confidential. Furthermore, participants were informed of their right to withdraw from the study at any time without this decision affecting the patient's care or treatment.

## Consent

Participation in the study was entirely voluntary. Written informed consent was obtained from all participants before data collection. They were given ample time to consider participation and could ask questions freely. Voluntariness was emphasized, and no form of pressure or persuasion was applied. Only those who provided written informed consent were included in the study. Confidentiality and anonymity were strictly maintained throughout the study process, and all collected data were stored securely and used solely for research purposes.

## Conflicts of Interest

The authors declare no conflicts of interest.

## Transparency Statement

The lead/Corresponding author (Neda Asadi) affirms that this manuscript is an honest, accurate, and transparent account of the study being reported; that no important aspects of the study have been omitted; and that any discrepancies from the study as planned have been explained.

## Data Availability

The data that support the findings of this study are available on request from the corresponding author. The data are not publicly available due to privacy or ethical restrictions. The datasets generated and/or analyzed during the current study are not publicly available [Due to the presence of identifiable participant information and confidentiality obligations outlined in the ethical protocol] but are available from the corresponding author on reasonable request.

## References

[1] N. Asadi , N. Khatoon Shoaei , and F. Salmani , “The Relationship Between Attitudes Towards Caring for Dying Patients and Self Compassion in ICU Nurses,” OMEGA ‐ Journal of Death and Dying 91, no. 4 (2025): 1879–1889. 10.1177/00302228231166537 37025044

[2] N. Asadi , Z. Royani , and S. Pourkhajoei , “Care Intertwined With Anxiety and Helplessness: The Experiences of ICU Nurses From COVID‐19 Disease's End of Life‐A Qualitative Study,” BMJ Open 15, no. 1 (2025): e087329. 10.1136/bmjopen-2024-087329 PMC11784321 39843377

[3] R. Patel , P. Huggard , and A. van Toledo, , “Occupational Stress and Burnout Among Surgeons in Fiji,” Frontiers in Public Health 5 (2017): 234533. 10.3389/fpubh.2017.00041 PMC5343003 28337433

[4] S. Riches and H. Smith , “Editorial: Taking a Break in the ‘New Normal’: Virtual Reality Relaxation for a Stressed Workforce,” Mental Health Review Journal 27, no. 2 (2022): 133–136.

[5] B. Salameh , J. Abdallah , S. A. Alkubati , and M. ALBashtawy , “Alarm Fatigue and Perceived Stress Among Critical Care Nurses in the Intensive Care Units: Palestinian Perspectives,” BMC Nursing 23, no. 1 (2024): 261. 10.1186/s12912-024-01897-x PMC11036661 38654236

[6] T. Moradi , F. Maghaminejad , and I. Azizi‐Fini , “Quality of Working Life of Nurses and Its Related Factors,” Nursing and Midwifery Studies 3, no. 2 (2014): e19450. PMC4228533 25414904

[7] W. Veling , B. Lestestuiver , M. Jongma , H. J. R. Hoenders , and C. van Driel, , “Virtual Reality Relaxation for Patients With a Psychiatric Disorder: Crossover Randomized Controlled Trial,” Journal of Medical Internet Research 23, no. 1 (2021): e17233. 10.2196/17233 PMC7846446 33448933

[8] J. L. Mosso Vázquez , A. Santander , J. L. Mosso, Jr. , K. Gao , B. Wiederhold , and M. D. Wiederhold , “Using Cybertherapy to Reduce Postoperative Anxiety in Cardiac Recovery Intensive Care Units,” Studies in Health and Technology Informatics 191 (2013): 88–92.

[9] J. W. H. M. Nijland , W. Veling , B. P. Lestestuiver , and C. M. G. Van Driel , “Virtual Reality Relaxation for Reducing Perceived Stress of Intensive Care Nurses During the COVID‐19 Pandemic,” Frontiers in Psychology 12 (2021): 706527. 10.3389/fpsyg.2021.706527 PMC8511693 34659021

[10] M. M. Meese , E. C. O'Hagan , and T. P. Chang , “Healthcare Provider Stress and Virtual Reality Simulation: A Scoping Review,” Simulation in Healthcare: The Journal of the Society for Simulation in Healthcare 16, no. 4 (2021): 268–274. 10.1097/SIH.0000000000000484 32890319

[11] M. Dehghan , S. Mokhtarabadi , E. Rashidi , E. Rahiminejad , and N. Asadi , “Correlation Between Professional Quality of Life and Alarm Fatigue Symptoms Among Intensive Care Unit Nurses,” Health Science Reports 6, no. 10 (2023): e1583. 10.1002/hsr2.1583 PMC10563168 37822846

[12] B. H. Stamm , The Concise ProQOL Manual (ProQOL. org, 2010). 2nd ed..

[13] M. Ghorji , Z. Keshavarz , A. Ebadi , and M. Nasiri , “Persian Translation and Psychometric Properties of Professional Quality of Life Scale (ProQOL) for Health Care Providers,” Journal of Mazandaran University of Medical Sciences 28, no. 163 (2018): 93–106.

[14] L. Bodet‐Contentin , M. Letourneur , and S. Ehrmann , “Virtual Reality During Work Breaks to Reduce Fatigue of Intensive Unit Caregivers: A Crossover, Pilot, Randomised Trial,” Australian Critical Care 36, no. 3 (2023): 345–349. 10.1016/j.aucc.2022.01.009 35246356

[15] S. Riches , P. Jeyarajaguru , L. Taylor , et al., “Virtual Reality Relaxation for People With Mental Health Conditions: A Systematic Review,” Social Psychiatry and Psychiatric Epidemiology 58, no. 7 (2023): 989–1007. 10.1007/s00127-022-02417-5 PMC9852806 36658261

[16] S. Fredericks , M. ElSayed , M. Hammad , et al., “Anxiety is Associated With Extraneous Cognitive Load During Teaching Using High‐Fidelity Clinical Simulation,” Medical Education Online 26, no. 1 (2021): 1994691. 10.1080/10872981.2021.1994691 PMC8555543 34710001

[17] F. Abasi and H. Farhadi , “The Effectiveness of Virtual Reality Package on Improving the Quality of Work Life and Organizational Vitality,” Tibbi‐i‐Kar 14, no. 4 (2023): 55–66.

[18] S. Y. Lee and J. Kang , “Effect of Virtual Reality Meditation on Sleep Quality of Intensive Care Unit Patients: A Randomised Controlled Trial,” Intensive and Critical Care Nursing 59 (2020): 102849. 10.1016/j.iccn.2020.102849 32241625

[19] S. Desai , C. Rath , N. Bhandarkar , G. Jape , and S. Rao , “Virtual Reality Experience to Relieve Stress, Burnout, Fatigue, and Anxiety in Healthcare Professionals: A Systematic Review,” Journal of Healthcare Management 70, no. 6 (2025): 416–434. 10.1097/JHM-D-24-00105 41217408

